# Developing a crisis model based on higher-order moments

**DOI:** 10.1016/j.heliyon.2022.e08896

**Published:** 2022-02-03

**Authors:** Vera Ivanyuk

**Affiliations:** a Department of Data Analysis and Machine Learning, Financial University under the Government of the Russian Federation, 125993, Moscow, Russia; b Department of Higher Mathematics, Bauman Moscow State Technical University, 105005, Moscow, Russia

**Keywords:** Time series, Statistical moment, Point estimation, Higher-order moments

## Abstract

In this paper, the goal is to create a model for detecting a crisis situation using the example of financial time series. An analysis of fixed and cumulative central and raw moments has been carried out. When conducting the analysis by higher-order moments, it has been shown that the moments record the presence of deviations and extreme values in the distribution of the series. Based on the higher-order moments, a crisis indicator has been proposed.

## 1 Introduction

In scientific and practical tasks, it is quite common to work with open systems, that is, systems that continuously interact with their environments. Such systems are characterized by the onset of a state in which the system parameters take threshold, critical values, while the degree of organization and order of the system decreases sharply and a return to the previous stable state becomes unlikely. This state is called a crisis. Thus, a crisis is an inevitable phase transition for any open self-regulating system. Quite often, the course or consequences of a crisis are a significant problem, for example, the consequences of an economic crisis or a sharp change of pressure in the gas dynamics, which can be followed by an explosion. At the same time, a crisis cannot be predicted. However, one can try to detect it at an early stage. The purpose of this study is to analyze time series based on higher-order moments and develop a crisis indicator that detects a crisis at an early stage.

The most significant contribution in the development of crisis models is Paul Krugman's model, for which he was awarded the Nobel Prize in Economics in 2008 [1]. P. Krugman finds the cause of the crisis in the inconsistency of monetary and exchange policy.

Worth noting is the paper by P. Cumperayot, R. Kouwenberg, who developed an econometric crisis model for exchange rates based on a multivariate extreme value approach [2]. This model includes three criteria: the exchange rate return ER, the exchange market pressure index EMP, and the real exchange market pressure index REMP. The authors estimate the measure of extremal association in the tail of the distribution of the sum of ER, EMP, and REMP and assess its currency crisis prediction performance.

Based on Extreme Value Analysis (EVA), currency crises were studied in the paper by Siregar, R., Pontines, V., & Rajan, R. [3]. In his work, Gander, J. P. [4] considers prices as random and independent variables and, based on Extreme value theory, draws conclusions about the behavior of stock market prices. In his paper [5], Kratz, M presents an overview of Univariate Extreme Value Theory (EVT) and suggests new approaches for modeling heavy-tailed distributions. In the paper [6], Schulze, N. describes Empirical quantiles, Regression quantiles and The Quantile Regression Model. In the article by Baur, D., & Schulze, N. [7] the role of systemic risk in normal and extreme market conditions is analyzed. The authors conclude that the financial crisis is significantly influenced by systemic risk. The authors Batten, J. A., Kinateder, H., Szilagyi, P. G., & Wagner, N. F. [8] assess the risk based on hedging. They note that the most common driver of hedge portfolio returns is the VIX. The authors Oleksiy Osiyevsky, Jim Dewald [9] assess the consequences of the crisis and claim that crisis has not only a negative effect on the economy but also a positive effect associated with economic recovery.

After analyzing the literature on modeling of crisis processes in the economy, we can conclude that many studies pay great attention to empirical research and concretization, which is expressed in specific values of changes in various indicators, such as volatility, the level of decrease in the asset values, and more. Notably, these studies are often not statistically justified. In the present work, it is proposed to develop a crisis indicator based on probability theory and mathematical statistics.

## 2 Literature

Time series analysis and estimation is a field of applied mathematics and is now widely used. For the study of time series, statistical methods were chosen – statistical moments of higher orders. A number of studies have already considered similar methods and models. For example, Jun, Ahn, and Kim [10] investigate the impact of the global financial crisis on the statistical properties of stock market indices, namely on the quarterly indicators of the International Monetary Fund. The authors develop an indicator of the onset of the crisis based on changes in these statistical properties, test it on 54 countries for 13 years and get fairly accurate results. Similar work is done in [11].

In many studies, researchers unanimously conclude that skewness and kurtosis provide more detailed information about the behavior of a time series than variance. So, in the article by Teng [12] a generalized detrended fluctuation analysis (DFA) is proposed to assess the complexity of such a dynamic system as the securities market. In the classical version of the DFA, the fluctuations of the series (volatility) are estimated based on variance, while the authors generalize the method using higher-order moments: the third-order (skewness) and the fourth-order (kurtosis) moments. This generalization allows us to study, in addition to volatility, such indicators as the skewness of the probability distribution of a real random variable relative to its value and the variability of the sharpness of the peak of the distribution, which, ultimately, allows us to create a more accurate picture of the behavior of the time series. Risk-neutral skewness and kurtosis work better than risk-neutral variance when using information about historical jumps to predict volatility. This conclusion is reached by some authors [13], their work explores the relationship between jump variations and risk-neutral moments when predicting volatility. A method is proposed that does not involve extrapolation when calculating risk-neutral moments, and it is documented that the risk-neutral skewness and kurtosis include the information content of historical jumps. Theoretical questions concerning the calculation of higher-order moments are considered in the paper [14]. The authors have proposed a method of multiscale high orders, and based on the calculations of skewness and kurtosis, this method is used to analyze and predict time series, including financial time series. The analysis was performed on artificial data, and then on data from the stock markets of several countries, and the method demonstrated high accuracy. High-order moments are also used in the work of Tang and Chen [15]. The authors conclude that the representation of high moments provides a new tool for diagnosing dynamic instability in time series. Similar questions are discussed in the article [16] – in this study, the predictability of realized skewness and realized kurtosis in relation to the volatility of the stock market is investigated. The authors extend the HAR-RV model of the volatility forecast by adding the realized skewness and kurtosis and come to the conclusion that this modification allows it to make the prediction more accurate. The HAR-RV method is not the only one that is generalized in this way. In [17], the authors generalized the Akaike information criterion to high-order statistics, such as skewness, kurtosis, and permutation entropy for analyzing the volatility of financial time series. Also, the analysis of financial time series using skewness and kurtosis is presented in [18].

Special attention is paid to the higher-order moments in the studies devoted to the risk in investing and the risk premium. So, the paper [19] addresses the volatility and skewness of the side effects of the risk premium in the stock market, where the risk premium is defined as the difference between risk-neutral and realized moments. More locally, using the example of gold and oil, this issue is discussed in the article [20]. This article analyzes the causal relationship not only between earnings and the overall variance of the gold and oil markets, but also jumps of volatility, skewness, and kurtosis. Higher-order moments are also used to predict the return on government bonds – this is the subject of the research [21]. This paper analyzes whether realized higher moments can predict sovereign bond return out of the sample using high-frequency data from the European bond market. As a result of the research, the authors conclude that the realized kurtosis is the dominant predictor of subsequent return among higher moments and other predictors. Can the skewness predict the excess return of a currency? This question is answered in the publication [22]. This article reveals the relationship between skewness and future returns in the foreign exchange market.

It is also worth paying attention to the works in which risk models and the construction of crisis indicators are studied. In Racicot, F.E. and Théoret, R. [23], financial risks and crises are assessed. The response of hedge funds to shocks is estimated based on higher-order moments. In earlier works, the authors Racicot, F.E. and Théoret, R. [24, 25, 26] estimate volatility, uncertainty and risk. The authors analyze the hedge fund returns and describe cyclical indicators. In later works, the authors Racicot, F.E. and Théoret, R. [27, 28] estimate the hedge fund strategies’ risks based on higher-order moments, and also use the Markov regime-switching process to assess asymmetry. In his writings, Taleb, N. [29] pays special attention to crisis processes and risk models.

In their work, Beaudry, P., Caglayan, M., Schiantarelli, F. [30] suggest uncertainty indicators. In the work of Baum, C. F., Caglayan, M., Ozkan, N. [31], the uncertainty of macroeconomic conditions is investigated. The paper of Calmès, C., Théoret, R., [32] explores how banks are responding to macroeconomic risk and uncertainty.

In the work of Auerbach, A. J., & Gorodnichenko, Y. [33], special states of market growth or decline are modeled using regime-switching models. In Bachman, R., Sims, E.R. [34], the impact of fiscal policy shocks on economic activity is analyzed. So in the above articles, based on higher-order moments, dynamics in time series are evaluated, crisis processes are analyzed and crisis indicators are suggested.

## 3 Method

A time series is a time-ordered sequence of values of some observed quantity (or a finite set of quantities). A time series consists of two elements: a discrete set of time values $T=\left\{t_{1},t_{2},\ldots \right\}$ in which the values of the observed quantity are read, and the numerical values of the quantity $X=X\left(T\right)=\left\{x_{1},x_{2},\ldots \right\}$ themselves, called the series levels [35, 36]. In this paper, the time series is considered as a random process with discrete time. In practice, the distribution of a random variable is unknown, there is only a set of values $x_{1},x_{2},\ldots ,x_{n}$. When trying to study some quantitative feature of a given sample, the problem arises of estimating the parameters that determine this distribution. The statistical estimate of an unknown parameter of a theoretical distribution is a function of the observed random variables. The study considers point estimates.

### 3.1 Statistical moments

One of the most interesting sets of statistics in assessing and predicting systemic crises are undoubtedly the moments of a random variable which are essentially its main characteristic point estimates. One of the advantages of using moments is the possibility of regulating their degree by changing the order. At the same time, as Taleb notes in [37], increasing the order of moments to eight not only raises their sensitivity but also leads to an increase in robustness and a decrease in the influence of external factors.

Moments are point estimates of the central tendency of some characteristic of a random variable.

To date, four characteristics reflected by moments are known, namely the central tendencies of the value (raw moment), the spread (standard deviation), the symmetry of the spread (skewness) and the thickness of the distribution tails. Importantly, higher-order moments (beyond the fourth order) replicate the properties of skewness (odd moments) and kurtosis (even moments) while having a higher sensitivity and robustness. Further in this paper, to determine the optimal order of higher moments, empirical estimation of the efficiency of moments from the 6th to the 10th order will be performed. Following that, the moments recognized as optimal will be subsequently used to develop a crisis indicator.

Also, when considering the particularities of moments, an important factor is their normalization, which can be done both according to arithmetic and geometric principles. This means that it will also be necessary to test both of these methods, with a subsequent evaluation of their efficiency and applicability for the indicator being developed.

In addition to the above, we will also need to assess the prospects of the regression estimation of moments of successive orders, since the tendencies of their linear coefficients may also be of considerable interest as a tool for early detection of crises.

Also, based on the Granger causality tests, the author will evaluate the predictive capabilities of the developed crisis indicator.

When developing the indicator, the following factors will be first taken into account:

− Sufficient or adjustable sensitivity.
− Sufficient or adjustable robustness.
− Mathematical simplicity (ease of formal understanding).
− Computational simplicity (costs of computer time).

#### 3.1.1 Averaging the point estimate

The main statistics of point estimates, depending on the scales used, can be expressed in three different ways of Kolmogorov averaging [38, 39]:

For a point estimation within an interval scale (for example, a non-normalized asset value), it is permissible to use arithmetic averaging of the following form only:

(1) $$\bar{X}=\frac{1}{n}\;\overset{n}{\underset{i=1}{\sum }}X_{i\;}$$

In this case, such statistics are generally considered unbiased, since they converge almost everywhere [40].

At the same time, for a point estimation within a ratio scale (for example, the asset return or the levelized cost), along [41] with the arithmetic averaging it is also acceptable to use a power averaging or geometric Kolmogorov-type averaging of the form:

(2) $$\bar{X}=\sqrt[{n}]{\overset{n}{\underset{i=1}{\prod }}X_{i\;}}\;or\;\bar{X}=\sqrt[{a}]{\overset{n}{\underset{i=1}{\sum }}{X_{i\;}}^{a}\;}\;$$

Since the statistics of all moments, except the raw moment, are biased point estimates, the use of unbiased values or at least consistent estimators may be required to improve the indicator readings. As a consistent (and in some cases, unbiased) estimator of the deviation characteristics of a random variable, geometric averaging of the following form can be used:

(3) $$\hat{\theta }=\sqrt[{a}]{\frac{n}{n-1,5}x^{a}}\;$$

Regarding the raw moment, it is worth noting individually that for the values described in the ratio scale, the statement [42] on the robustness of the majorant of the power mean is valid. The stability of estimates for unreduced values of the form θ=E(x) may be purposefully increased by reducing the form $\sqrt[{a}]{\frac{n}{n-1,5}x^{a}}$ for values a>1, or decreased for a<1.

Since the higher moments represent the characteristics of the deviation of a random variable (even ones characterize a modular variable, and odd ones pertain to a vector variable), this method is applicable to obtain their respective unbiased estimates.

Some of the proven unbiased estimation methods are also suitable for obtaining unbiased estimates of moments:

− for variance: $s^{2}=\frac{1}{n-1}\overset{n}{\underset{i=1}{\sum }}{\left(X_{i}-\bar{X}\right)}^{2}\;$ or $\;M_{2}=\frac{n}{n-1}\mu_{2}$
− for skewness: $M_{3}=\frac{n^{2}}{\left(n-1\right)\left(n-2\right)}\mu_{3}$
− for kurtosis: $M_{4}=\frac{n\left(n^{2}-2n+3\right)\mu_{4}-3n\left(2n-3\right){\mu_{2}}^{2}}{\left(n-1\right)\left(n-2\right)\left(n-3\right)}$

#### 3.1.2 Classical moments

Classical moments include moments from the first to the fourth order, having the following characteristics:

− The raw moment is an unbiased, strongly consistent point estimator of the mathematical expectation of a random variable and is calculated as:

(4) $$\;\nu_{1}=\bar{X}=\frac{1}{n}\;\overset{n}{\underset{i=1}{\sum }}X_{i\;}$$

− The second-order central moment (variance) is a biased, weakly consistent estimator of the mathematical expectation of the squared deviations of a random variable and is calculated as:

(5) $$\;\mu_{2}=D=\sigma^{2}=\frac{1}{n}\;\overset{n}{\underset{i=1}{\sum }}{\left(X_{i}-\bar{X}\right)}^{2}$$

− The third-order central moment (skewness) is a biased, weakly consistent estimator of the mathematical expectation of the lopsidedness of the distribution tails of a random variable and is calculated as:

(6) $$\mu_{3}=\frac{1}{n}\;\overset{n}{\underset{i=1}{\sum }}{\left(X_{i}-\bar{X}\right)}^{3}$$

− The fourth-order central moment (kurtosis) is a biased, weakly consistent estimator of the mathematical expectation of the width of the distribution tails of a random variable and is calculated as:

(7) $$\mu_{4}=\frac{1}{n}\;\overset{n}{\underset{i=1}{\sum }}{\left(X_{i}-\bar{X}\right)}^{4}$$

− The general formula for calculating the subsequent central moments is uniform and returns the characteristics of skewness and spread, respectively, for odd and even orders:

(8) $$\;\mu_{k}=\frac{1}{n}\overset{n}{\underset{i=1}{\sum }}{\left(X_{i}-\bar{X}\right)}^{k}$$

Since the moments themselves are already relative values, a geometric or power-law normalization based on Kolmogorov's method can be used to bring them to a unified dimensionless scale.

#### 3.1.3 Arithmetic and Power-law normalization

The power-law first-degree (arithmetic) normalization to the relative scale of standard deviations is the most generally accepted. Based on the z-score, it allows bringing the moments of the i-st order to the standard form:

(9) $$\beta_{i}=E\left[{\left(\frac{X-\nu_{1}}{\sqrt{\sigma^{2}}}\right)}^{i}\right]$$

To obtain a standardized dimensionless value of the 3rd-order moment, arithmetic normalization is also used: $\gamma_{1}=\frac{\mu_{3}}{\sigma^{3}}$. The unbiased estimator of the skewness, respectively, will have the form:

(10) $$\hat{\gamma_{1}}=\frac{\sqrt{n\left(n-1\right)}}{n-2}\left(\frac{\mu_{3}}{{\mu_{2}}^{\frac{3}{2}}}\right)\;$$

By analogy with skewness, standardization is carried out by arithmetic normalization of the 4th-order moment with an additional bias:

(11) $$\gamma_{2}=\frac{\mu_{4}}{\sigma^{4}}-3$$

and its unbiased estimator is:

(12) $$\hat{\gamma_{2}}=\frac{n^{2}-1}{\left(n-2\right)\left(n-3\right)}\left(\frac{{\hat{\mu }}_{4}}{{{\hat{\mu }}_{2}}^{2}}-3+\frac{6}{n+1}\right)$$

Similarly to the arithmetic approach, Kolmogorov's method of power-law averaging can be applied to the ratio scale. Then, what is important, unlike the previous one, it has controllable robustness. Thus, according to the majority rule of power means, with an increase in the averaging degree for values normalized to one, both the magnitude and robustness of the point estimate increases. In other words: the majorant of the power mean is less robust from above and more robust from below. Similarly to the aforesaid, normalization of the following form is also acceptable:

(13) $$\bar{\bar{\gamma_{i}}}=\sqrt[{i}]{E\left[\frac{{\left(Χ-\nu_{1}\right)}^{i}}{\sigma^{i}}\right]}$$

Accordingly, a consistent estimator of higher-order moments can be presented, in contrast to the arithmetic method described above, in a simpler form, as:

(14) $$\hat{\gamma_{i}}=\sqrt[{i}]{E\left[\frac{n}{n-1,5}\frac{{\left(Χ-\nu_{1}\right)}^{i}}{\sigma^{i}}\right]}\;$$

At the same time, an increase in the averaging degree makes it possible to achieve the necessary robustness of the estimator.

With this normalization method, it is important to note the fundamental role of the central moment, since its order affects the overall robustness. At the same time, the interval scale allows the reduction of average values using not only an arithmetical but also a geometrical method as in:

(15) $$\bar{\bar{\nu_{i}}}=\sqrt[{i}]{E\left[sgn\left(X\right)*{Χ}^{i}\right]}$$

and, accordingly, the calculation of higher-order robust moments as

(16) $$\bar{\bar{\mu_{i}}}=\sqrt[{i}]{Μ\left[{\left(Χ-\bar{\bar{\nu_{i}}}\right)}^{i}\right]}$$

When analyzing time series, three types of raw and central moments are used, which differ in temporal characteristics.

The total moment is the moment of the entire time series. It has a numeric value and is calculated for the whole series. It is the only finite point estimate.

The fixed moment is a moment that corresponds to a certain time interval. It has a numeric value, calculated for a series $X^{*}\;$ that is a subset of the original series X. It is a multiple moving estimate.

The cumulative moment is the moment corresponding to the time interval from the beginning of the series. It has a numeric value, calculated for a series $X_{0}-X^{*}$ that is a subset of the original series X. It is a multiple moving estimate.

When conducting the study, it will be necessary to evaluate the relationship of the sensitivity and robustness of higher-order moments both to the degree of averaging and to different time intervals of the series $\;X^{*}$.

### 3.2 Computational complexity

To assess the computational complexity, we will use the clock latencies for the most common microprocessor architectures, given in [43]. At the same time, we will take into account the possibility of up to eight simultaneous vector operations of the same type, considering the costs of data transport to be identical (Table 1).

**Table 1:** Computational complexity.

Execution latencies, typical x 10	AMD	Core 245 nm	Sandy Bridge	Avg	Eff
floating point addition (ADD)	40	30	30	33,3	33,3
floating point multiplication (MUL)	40	50	30	40	40
floating point division, square root (DIV)	110	160	110	126,6	126,6
floating point vector addition (VADD)	40	30	30	33,3 (8)	12,5
floating point vector multiplication (VMUL)	40	50	50	46,6 (8)	17,5

Let us calculate the rounded computational costs for ten operations of computing unreduced fixed moments with a five-day interval $X^{*}$ (for other intervals, the costs increase by a multiple), taking into account the possibilities of vector optimization of sequential addition and multiplication operations:

$$1.\;\nu_{1}=\frac{1}{n}\;\overset{n}{\underset{i=1}{\sum }}X_{i\;};\;VADD+DIV\approx 160$$

$$2.\;\mu_{2}=\frac{1}{n}\;\overset{n}{\underset{i=1}{\sum }}{\left(X_{i}-\bar{X}\right)}^{2};ADD*5+MUL*5+VADD+DIV\approx 426$$

$$3.\;\gamma_{1}=\frac{1}{n}\;\overset{n}{\underset{i=1}{\sum }}{\left(X_{i}-\bar{X}\right)}^{3};\left(ADD+VMUL\right)*5+VADD+DIV\approx 559$$

$$4.\;\gamma_{2}=\frac{1}{n}\;\overset{n}{\underset{i=1}{\sum }}{\left(X_{i}-\bar{X}\right)}^{4};\;\left(ADD+VMUL\right)*5+VADD+DIV\approx 559$$

$$5.\;\gamma_{k<7}=\frac{1}{n}\overset{n}{\underset{i=1}{\sum }}{\left(X_{i}-\bar{X}\right)}^{k};\;\left(ADD+VMUL\right)*5+VADD+DIV\approx 559$$

$$6.\;\gamma_{6<k<14}=\frac{1}{n}\overset{n}{\underset{i=1}{\sum }}{\left(X_{i}-\bar{X}\right)}^{k};\;\left(ADD+VMUL*2\right)*5+VADD*2+DIV\approx 826$$

In this case, one should pay attention to the fact that with proper optimization [44] of the vector computing, gammas from the first to the sixth order (moments from the third to the eighth) have identical computational complexity. Thus, in terms of computing speed, the higher-order moments are more computationally advantageous compared to the lower-order moments.

Next, we estimate the computational complexity of the reduced moments:

Unbiased efficient consistent z-score:

$$7.\;\hat{\gamma_{1}}=\frac{\sqrt{n\left(n-1\right)}}{n-2}\left(\frac{\frac{1}{n}\;\sum_{i=1}^{n}{\left(X_{i}-\bar{X}\right)}^{3}}{\frac{1}{\;n}\;\sum_{i=1}^{n}{\left(X_{i}-\bar{X}\right)}^{2\frac{3}{2}}}\right);\;ADD*2+MUL*3+DIV*9+5*\left(VADD+VMUL\right)+5*\left(VADD+VMUL*2\right)\approx 2361$$

$$8.\;\hat{\gamma_{2}}=\frac{n^{2}-1}{\left(n-2\right)\left(n-3\right)}\left(\frac{\frac{n\left(n^{2}-2n+3\right)\frac{1}{n}\;\sum_{i=1}^{n}{\left(X_{i}-\bar{X}\right)}^{4}-3n\left(2n-3\right)\frac{1}{n}\;\sum_{i=1}^{n}{\left(X_{i}-\bar{X}\right)}^{2^{2}}}{\left(n-1\right)\left(n-2\right)\left(n-3\right)}}{{\left(\frac{n}{n-1}\frac{1}{n}\sum_{i=1}^{n}{\left(X_{i}-\bar{X}\right)}^{2}\right)}^{2}}-3+\frac{6}{n+1}\right)\;MUL*10+DIV*8+ADD*13+5*\left(VADD+VMUL\right)+10*\left(VADD+2*VMUL\right)\approx 3510$$

Unbiased consistent z-score:

$$9.\;\hat{\gamma_{i<7}}=\sqrt[{i}]{\frac{1}{n}\overset{n}{\underset{i=1}{\sum }}\left(\frac{n}{n-1,5}\frac{{\left(Χ-\bar{Χ}\right)}^{i}}{\sigma^{i}}\right)\;};\;ADD+DIV*8+MUL+\left(VADD+VMUL*2\right)*5\approx 1719$$

Unbiased consistent estimator:

$$10.\;\bar{\bar{\gamma_{i<7}}}=\sqrt[{i}]{\frac{1}{n}\overset{n}{\underset{i=1}{\sum }}\left(\frac{n}{n-1,5}{\left(Χ-\bar{Χ}\right)}^{i}\right)};\;ADD+DIV*3+MUL+\left(VADD+VMUL\right)*5\approx 853$$

To evaluate promising methods for detecting a crisis by higher-order moments, we will compile a comparative table rating the desired capabilities on a ten-point scale (Table 2).

**Table 2:** Comparative table rating.

Estimator property	Consistent estimator	Unbiased consistent Z-score	Unbiased efficient Z-score
Adjustable sensitivity	10	5	0
Adjustable robustness	10	5	0
Mathematical complexity	9	6	1
Computational complexity	10	5	1
Total	39	21	2

For an empirical estimator, we will discard the worst-case variant of a point estimate. Next, it is necessary to carry out an empirical estimation of the higher-order moments as crisis indicators for at least five of the most well-known economic crises.

## 4 Result

### 4.1 Empirical estimation of higher-order moments

**Figure 1:**
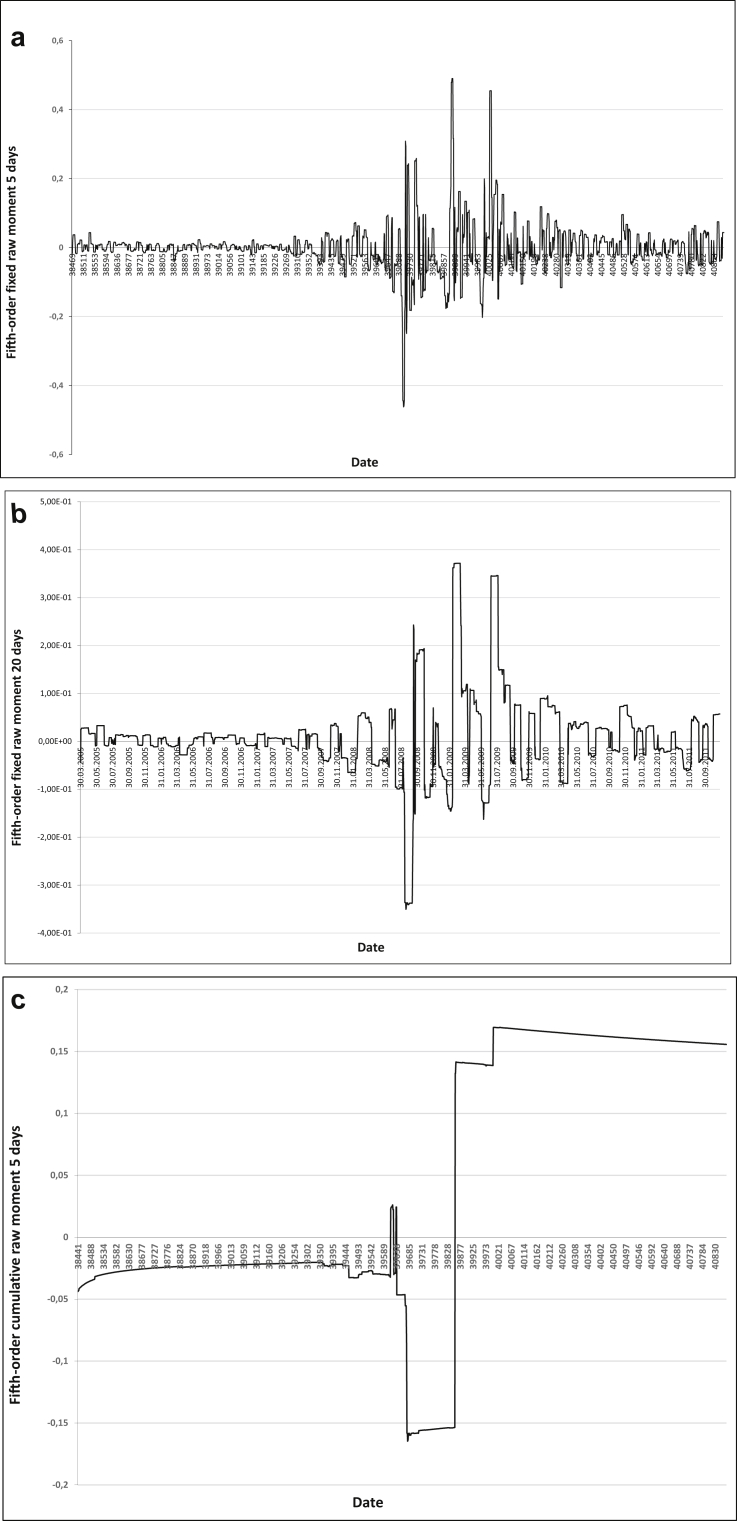
AIG insurance company assets a) Fifth-order fixed raw moment 5 days, b) Fifth-order fixed raw moment 20 days, c) Fifth-order cumulative raw moment 5 days.

**Figure 2:**
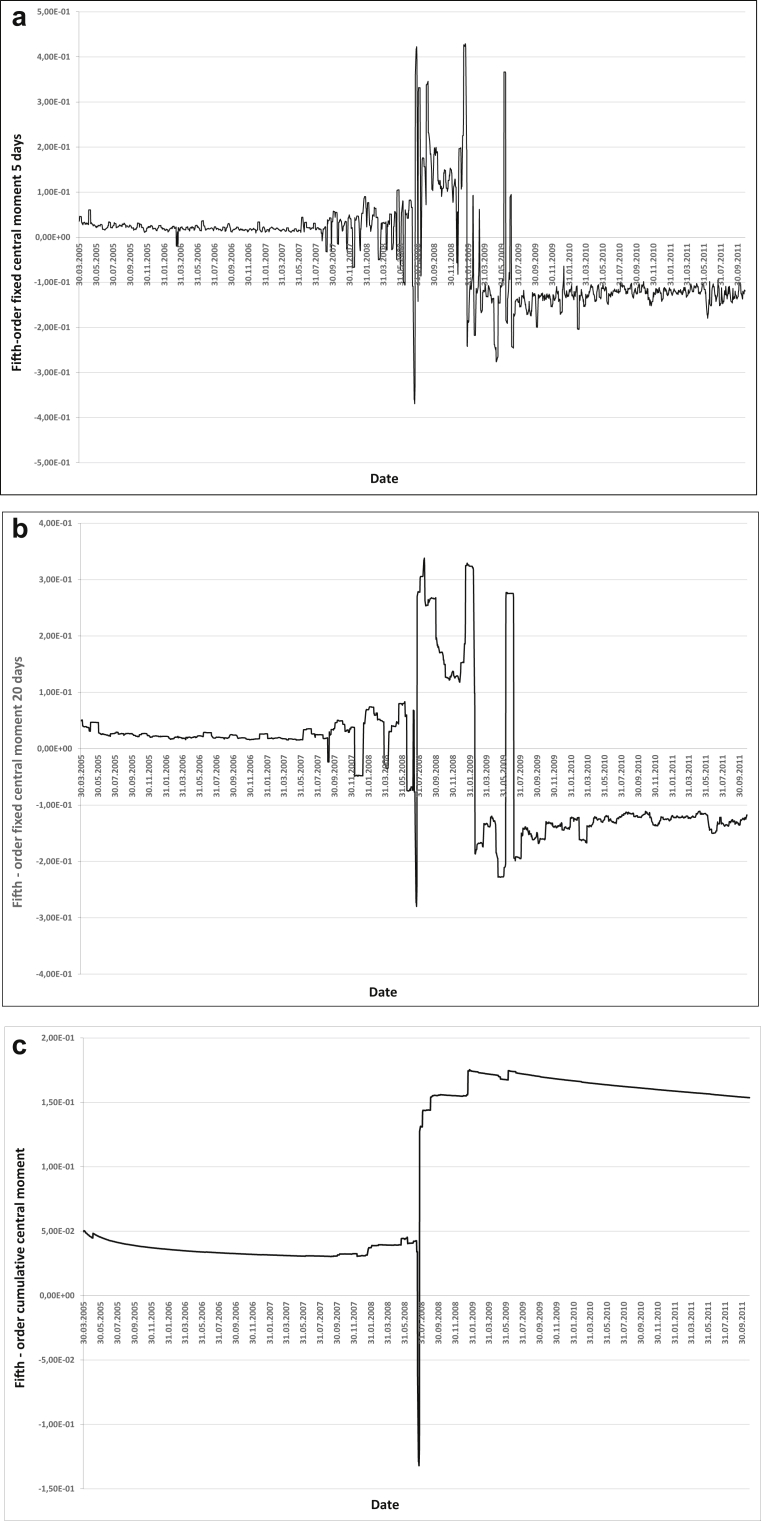
AIG insurance company assets a) Fifth-order fixed central moment 5 days, b) Fifth-order fixed central moment 20 days, c) Fifth-order cumulative central moment.

Let us conduct an intuitive assessment of 5- and 20-day fixed moments and cumulative central and raw moments. For the assessment, we use graphs of the reduced raw and central moments of the fifth order which are regarded as having moderate robustness properties (Figures 1 and 2).

Obviously, with an increase in the sample size, the robustness of the moment increases as well, with the cumulative moment being the most robust one.

Relative quantitative assessment will be carried out by counting the number of extrema of 5-, 10-, 15- and 20-day fixed and cumulative central and raw moments (Table 3).

**Table 3:** Quantitative assessment of robustness.

Fifth-order moment	Number of extrema	Relative robustness	Average robustness score
5-day raw moment	818	1	1
5-day central moment	814	1	
10-day raw moment	809	1,01	1,015
10-day central moment	800	1,02	
15-day raw moment	828	0,99	1,005
15-day central moment	801	1,02	
20-day raw moment	826	0,99	1,005
20-day central moment	805	1,02	
Raw cumulative moment	149	5,49	5,435
Central cumulative moment	152	5,38	

According to the quantitative assessment, with an increase in the time length of the fixed moment from 5 to 20 days, there is practically no significant increase in robustness, while the cumulative moment appears to be the most robust of all the estimated ones. This is the moment we will use to assess robustness further in the study.

**Figure 3:**
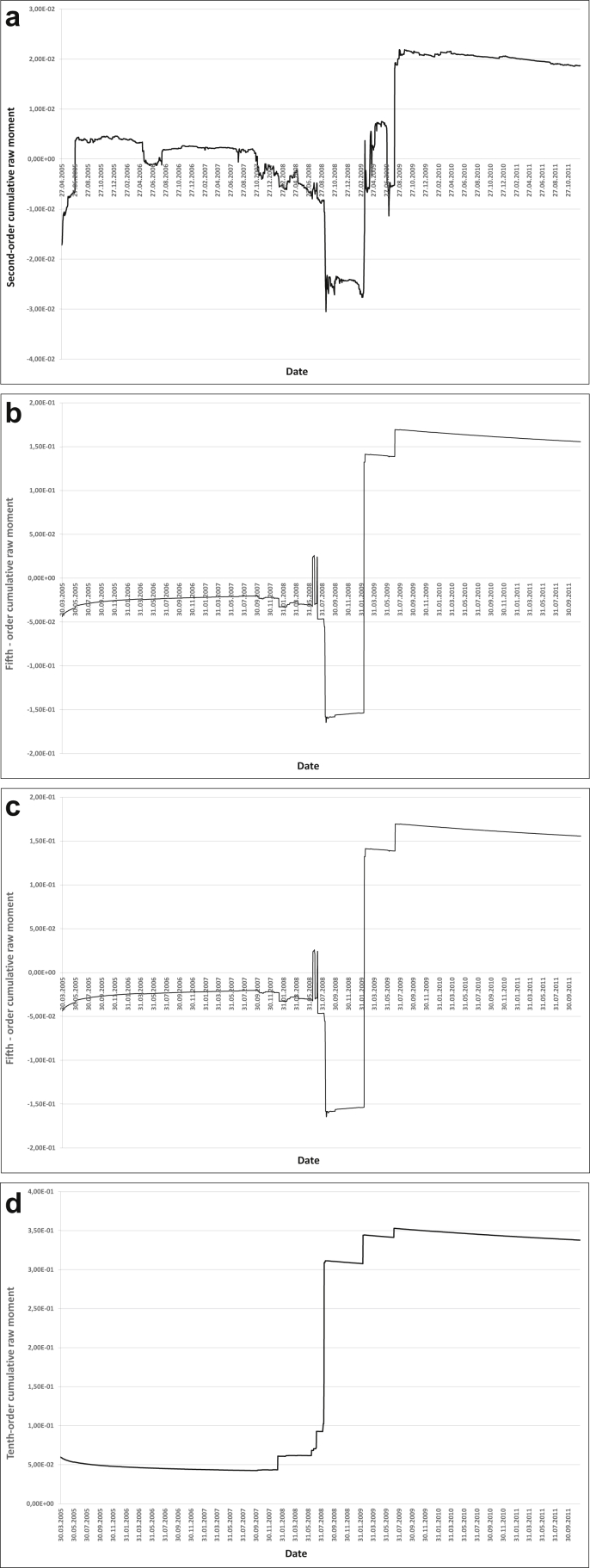
AIG insurance company assets a) Second-order cumulative raw moment, b) Fifth-order cumulative raw moment, c) Ninth-order cumulative raw moment, d) Tenth-order cumulative raw moment.

**Figure 4:**
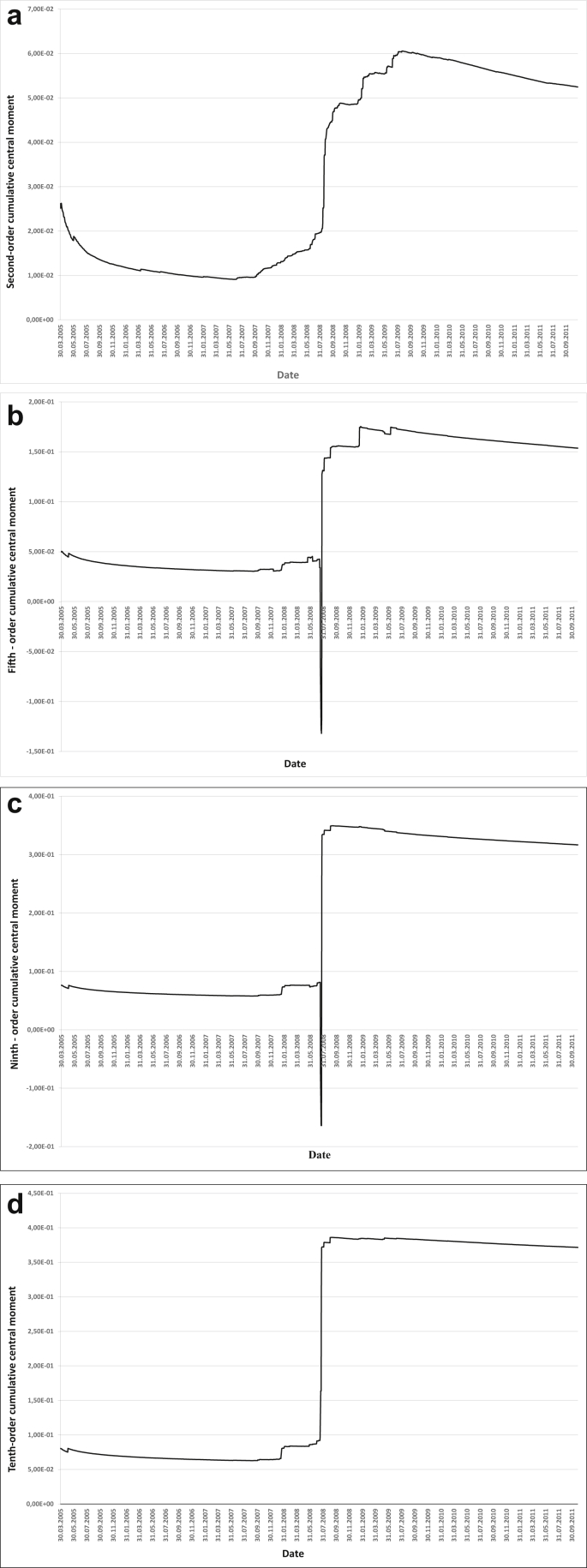
AIG insurance company assets a) Second-order cumulative central moment, b) Fifth-order cumulative central moment, c) Ninth-order cumulative central moment, d) Tenth-order cumulative central moment.

We will conduct an intuitive and quantitative assessment of robustness for cumulative central and raw moments of the 2nd, 5th, 9th and 10th orders. For an intuitive assessment, we use the graphs of the 2nd, 5th, 9th and 10th reduced raw and central cumulative moments (Figures 3 and 4).

Quantitative assessment will be carried out by counting the number of extrema of cumulative central and raw moments of the 2nd order, as well as 6th to the 10th orders (Table 4).

**Table 4:** Quantitative assessment of robustness.

Cumulative moment	Number of extrema	Relative robustness	Average robustness score
2nd-order raw moment	335	1	1
2nd-order central moment	185	1	
6th-order raw moment	52	6,44	3,855
6th-order central moment	146	1,27	
7th-order raw moment	40	8,38	5,615
7th-order central moment	65	2,85	
8th-order raw moment	29	11,55	6,865
8th-order central moment	85	2,18	
9th-order raw moment	27	12,41	7,830
9th-order central moment	57	3,25	
10th-order raw moment	35	9,57	6,085
10th-order central moment	71	2,6	

It is obvious that with an increase in the order of the moment, its robustness increases as well, with the cumulative moments of higher orders being the most robust ones.

### 4.2 Development of a crisis indicator

With an intuitive analysis of higher-order moment graphs, it becomes apparent that the change in the return regime is reflected by a significant change in the partial derivative of the moment, which looks like a nearly vertical increment on the graph.

**Figure 5:**
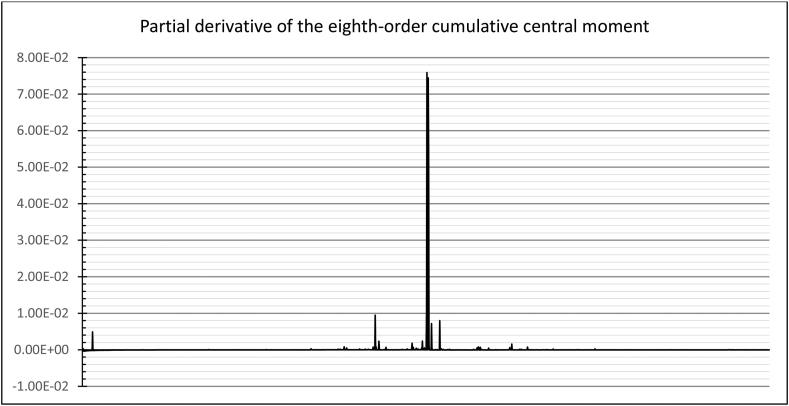
Partial derivative of the eighth-order central moment.

We will estimate the partial derivative of the eighth-order central moment as the most robust indicator of the rapidly computable ones (Figure 5).

As we can see, the moments of crisis growth of the partial derivative are by one order of magnitude greater than the usual pre-crisis and post-crisis outliers.

It is important to note that at the initial section of the graph there is an inevitable spike in the value of the derivative corresponding to the period of accumulation of the standard deviation values. Depending on the necessary reliability of the crisis detection, the initial section of the time series, lasting from 23 (2σ) to 371 (3σ) sample elements, can be considered the spike area.

To form the indicator, we use the suprema of the previous derivative values and the Heaviside function as a YES/NO formalizer, while, depending on the desired accuracy degree of the crisis detection, the sample size of subsets $X^{*}$ and $X_{0}^{*}$ may have an arbitrary value corresponding to the estimated number of z-values of the standard deviation of the statistical population. In this case, the sample $X_{0}^{*}$ reflects the accumulation of the standard deviation of the time series in the initial period and is disregarded, while the sample $X^{*}$ covers a certain number of preceding crisis-free days.

(17) $$P=\text{H}\left(\frac{\partial \gamma_{k}}{\partial t}-{\text{sup}}_{X^{*}}\frac{\partial \gamma_{k}}{\partial t}\right)$$

(18) $$C=\text{H}\left(\frac{\partial \gamma_{k}}{\partial t}-z\left({\text{sup}}_{X^{*}}\frac{\partial \gamma_{k}}{\partial t}\right)\right)$$

**Figure 6:**
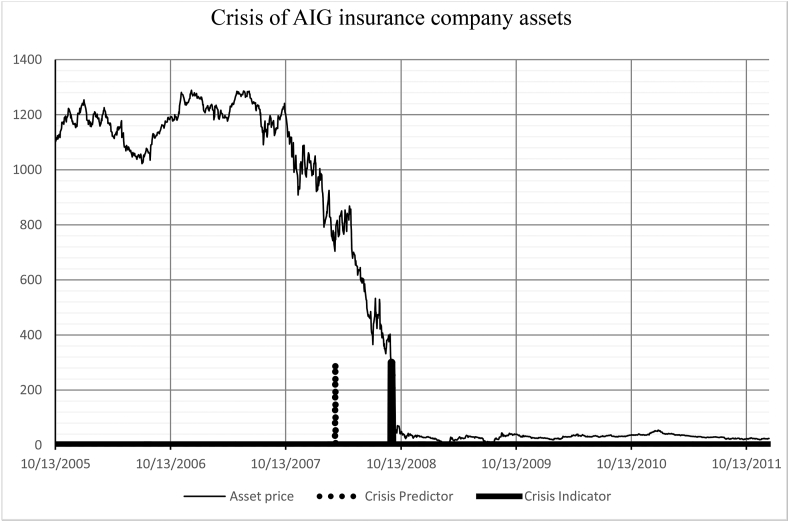
Identification of the 2008 crisis. American International Group (30.03.2005–23.12.2011).
− 01.03.2005–30.12.2011 – Bank of America (Figure 7).

Let us test the indicator on historical data. Consider the time series of the following periods:

− 30.03.2005–23.12.2011 – American International Group (Figure 6).

**Figure 7:**
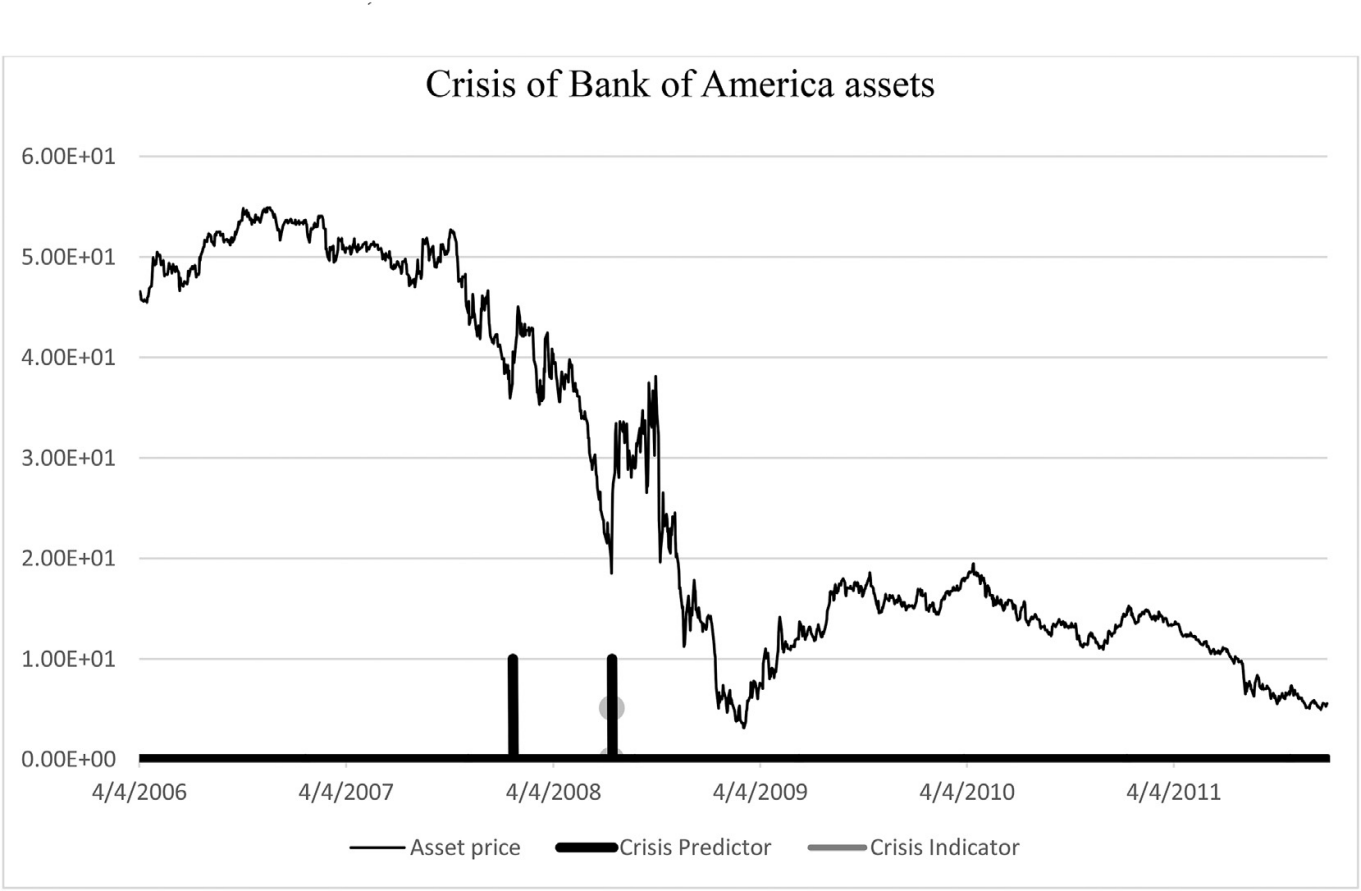
Identification of the 2008 crisis. Bank of America (01.03.2005–30.12.2011).
− 08.04.2009–19.11.2021 – USD/RUB TOM (Figure 8).

**Figure 8:**
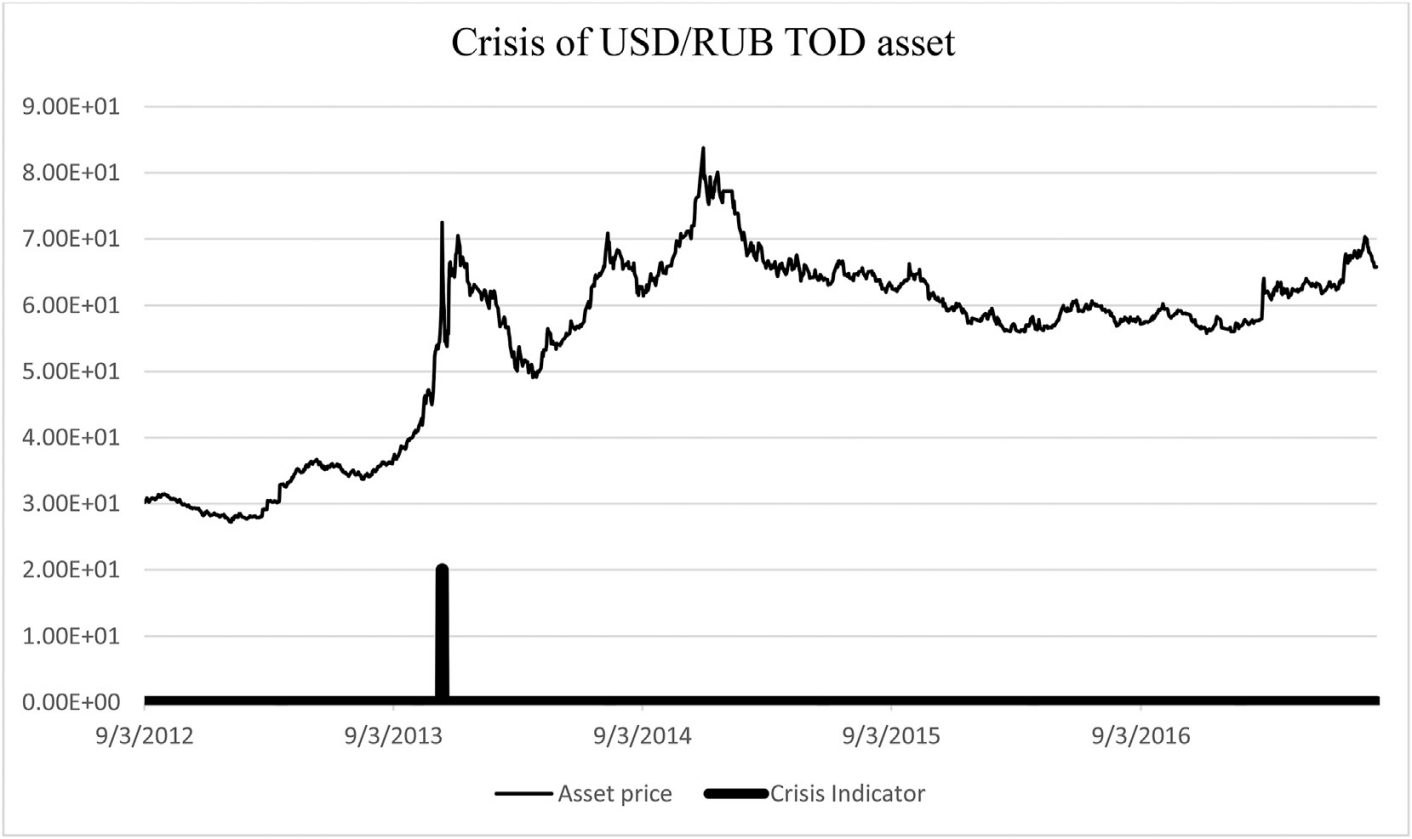
Identification of the Russian currency crisis of 2014. USD/RUB (08.04.2009–19.11.2021).

## 5 Conclusion

In the article, the following tasks have been set and solved:

− analysis of 5- and 20 day fixed and cumulative central and raw moments; analysis of cumulative central and raw moments of the 2nd, 5th, 9th and 10th orders.
− identification of the onset of a crisis situation using the developed crisis indicator.

When analyzing by higher-order moments, it was shown that with an increase in the sample size, the robustness of the moment increases. The cumulative moment proved to be the most robust one.

As the order of the moment increases, its robustness increases as well. The most robust ones are cumulative moments of higher orders.

## Declarations

### Author contribution statement

Vera Ivanyuk: Conceived and designed the experiments; Performed the experiments; Analyzed and interpreted the data; Contributed reagents, materials, analysis tools or data; Wrote the paper.

### Funding statement

This research did not receive any specific grant from funding agencies in the public, commercial, or not-for-profit sectors.

### Data availability statement

Data included in article/supplementary material/referenced in article.

### Declaration of interests statement

The authors declare no conflict of interest.

### Additional information

No additional information is available for this paper.
